# The Role of the Immune Response in Merkel Cell Carcinoma

**DOI:** 10.3390/cancers5010234

**Published:** 2013-02-28

**Authors:** Pierre L. Triozzi, Anthony P. Fernandez

**Affiliations:** 1 Taussig Cancer Institute, Cleveland Clinic Foundation, 9500 Euclid Avenue, Cleveland, OH 44195, USA; 2 Departments of Dermatology and Anatomic Pathology, Cleveland Clinic Foundation, 9500 Euclid Avenue, Cleveland, OH 44195, USA; E-Mail: fernana6@ccf.org

**Keywords:** merkel cell polyomavirus, immune suppression, tumor infiltrating lymphocytes, regulatory T cells, immunotherapy

## Abstract

Merkel cell carcinoma (MCC) is an aggressive neuroendocrine skin cancer. The Merkel cell polyomavirus (MCPyV) is implicated in its pathogenesis. Immune mechanisms are also implicated. Patients who are immunosuppressed have an increased risk. There is evidence that high intratumoral T-cell counts and immune transcripts are associated with favorable survival. Spontaneous regressions implicate immune effector mechanisms. Immunogenicity is also supported by observation of autoimmune paraneoplastic syndromes. Case reports suggest that immune modulation, including reduction of immune suppression, can result in tumor regression. The relationships between MCPyV infection, the immune response, and clinical outcome, however, remain poorly understood. Circulating antibodies against MCPyV antigens are present in most individuals. MCPyV-reactive T cells have been detected in both MCC patients and control subjects. High intratumoral T-cell counts are also associated with favorable survival in MCPyV-negative MCC. That the immune system plays a central role in preventing and controlling MCC is supported by several observations. MCCs often develop, however, despite the presence of humoral and cellular immune responses. A better understanding on how MCPyV and MCC evade the immune response will be necessary to develop effective immunotherapies.

## 1. Introduction

Merkel cell carcinoma (MCC) is a rare, neuroendocrine carcinoma that originates in the skin from cells that normally function as mechanoreceptors. It is an aggressive neoplasm that has a predilection for local recurrence, regional metastasis to lymph nodes, and distant metastasis to lung, liver, bone, and brain. MCC arises primarily on sun-exposed areas, with the head and neck being most commonly affected. Older, lighter-skinned individuals with a history of significant sun exposure are at the highest risk [1]. Approximately 80% of MCCs harbor the Merkel cell polyomavirus (MCPyV), a small, non-enveloped, double-stranded DNA virus that appears to play a role in its molecular pathogenesis [2]. As with most cutaneous malignancies, surgery is the mainstay of therapy. Although definitive evidence is lacking, there are data suggesting that radiation to the primary site and involved regional lymph nodes can improve locoregional control [3]. There is, however, little evidence that radiation affects overall survival. The chemotherapeutics usually administered are those used to treat small-cell lung carcinoma, also a high-grade neuroendocrine carcinoma. Whether chemotherapy in the setting of metastatic disease improves survival is not established [4,5]. Furthermore, the retrospective analyses that have been conducted do not indicate that adjuvant chemotherapy improves overall survival [6,7].

Although named after the cancer in which it was discovered, MCPyV is prevalent in normal skin, being detected in over 90% of forehead swabs from patients with MCC and in 60 to 80% of skin swabs from healthy volunteers [8,9]. Similar to other polyomaviruses, MCPyV encodes two major families of genes, early and late. The early genes encode the T antigens, proteins that promote replication of viral DNA. The largest of the T antigens (LT-Ag) is persistently expressed in a truncated form in MCC tumors and likely promotes cell division by inhibiting the retinoblastoma tumor suppressor protein. At the same time, the truncated LT-Ag no longer induces lytic viral replication that would be lethal to a cancer cell. The small T antigen (ST-Ag), which shares the first 78 amino acids with LT-Ag, is also expressed in MCC tumors. It is thought to inhibit the tumor suppressor protein phosphatase 2A through its unique c-terminal domain [10,11]. The late genes encode proteins that form the viral capsid. Although required for initial infection, capsid expression cannot be detected in MCC tumors and, thus, does not appear to play a role in tumor persistence [12]. Individuals with MCPyV-positive MCC may have more favorable outcome than those with MCPyV-negative MCC. Mutations in the *TP53* tumor suppressor gene, which are associated with a poor prognosis and resistance to therapy of many cancers, are more frequent in MCPyV-negative MCCs [13].

That tumor immune surveillance exists in humans is supported by a number of clinical observations, including the increased risk of tumor development in immunosuppressed patients, instances of spontaneous tumor regression, and appearance of tumor-reactive antibodies and T cells. Several clinical observations implicate the immune response in the development and progression of MCC. Herein we review the role of the immune response on the incidence and course of MCC, including the role of MCPyV-specific immune responses. The potential for immune modulating therapies is also reviewed.

## 2. Incidence

Immunosuppression caused by either therapy or by primary or secondary immunodeficiencies is associated with an increased risk of many malignancies. Although over 90% of MCC patients do not have any overt immune dysfunction, the association between MCC and immunosuppression is well established. It has been estimated that the risk of MCC increases 15-fold over the general population in immunosuppressed patients [14]. In organ transplant patients receiving immunosuppressives, skin cancers are the most common cancers that develop. Although MCC accounts for less than 5% of all skin cancers in organ transplant recipients, these patients have a relative risk for the development of MCC estimated to be approximately 10-fold higher than the general population [15]. It was noted in a review of 41 cases of MCC reported to the Cincinnati Transplant Tumor Registry (now called the Israel Penn International Transplant Tumor Registry) that patients with MCC in the setting of organ transplantation tended to be younger [16]. The mean age at diagnosis was 53 (range 33–78) years, and 29% of recipients were less than 50 years old. The tumor appeared from approximately 6 months to 24 years after the transplant with the mean being approximately 8 years. The site of development was similar to that seen in the non-transplant population, with the head and neck being the most commonly affected. Approximately half of the transplant recipients with MCC had other malignancies, the great majority of which (91%) were other skin cancers. That 68% of patients in this case series developed lymph node metastases and 56% died of their malignancies supported the possibility that MCC in the transplant setting was more aggressive that in the general population. Review of the Finnish Cancer Registry also suggested that patients with MCC in the organ transplant setting are younger and manifest aggressive clinical courses [17]. An association with autoimmune disease was also raised. Only one of the three MCC cases reported in this series was MCPyV positive.

A 30-fold increased incidence of MCC in patients with chronic lymphatic leukemia (CLL) has been reported; an increased incidence has also been observed in patients with multiple myeloma and non-Hodgkin lymphoma (NHL) [18]. Cell-mediated immune responses are compromised in the setting of lymphoid malignancies, and these patients may have a higher propensity for colonization with MCPyV, thus influencing the subsequent development of MCC. Patients with lymphoid malignancies also often receive T-cell-suppressive therapies that may influence infection with and behavior of MCPyV and ultimately the course of MCC. Review of cases in the Surveillance, Epidemiology, and End Results program found that overall survival among patients with MCC with a history of CLL or NHL was worse than expected [19]. MCC cause-specific survival was also worse than expected for patients with a history of CLL, but no difference was observed for NHL. Whether MCC predisposes to hematologic malignancy is not known. Epidemiological data, however, do indicate an increase in hematological malignancies, primarily lymphoid, diagnosed after diagnosis of MCC [18]. Interestingly, MCPyV is only rarely detected in tissue from these hematologic malignancies, and then only at low viral loads [20,21]. It may be an immune aberration leads to both malignancies or be merely the result of increased surveillance of these patients. The incidence of MCC is also increased in patients treated for autoimmune disease and in the setting of HIV infection [22,23].

## 3. Clinical Course

### 3.1. Tumor Infiltrating Cells

**Figure 1:**
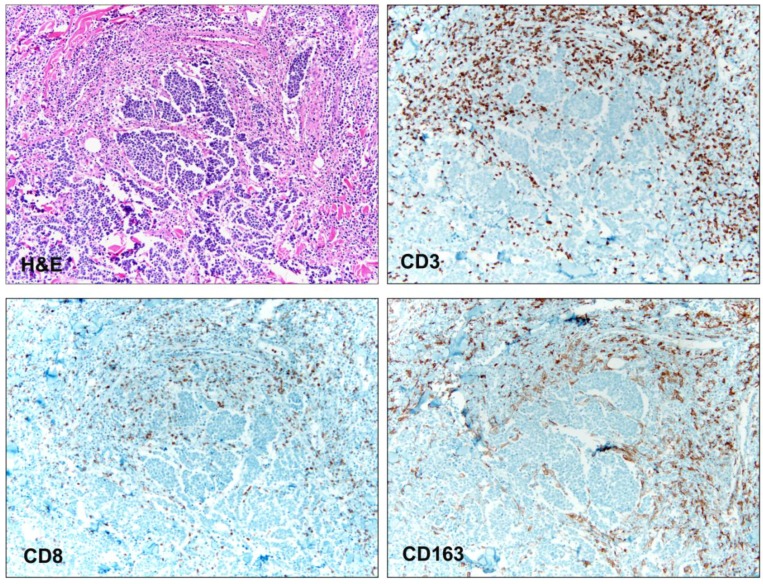
Photomicrogaphs (10× magnification) of a cutaneous Merkel cell carcinoma that is fragmented (H&E) and associated with an inflammatory infiltrate. Immunohistochemical stains reveal presence of tumor infiltrating lymphocytes (CD3, CD8) and tumor infiltrating macrophages (CD163).

**Figure 2:**
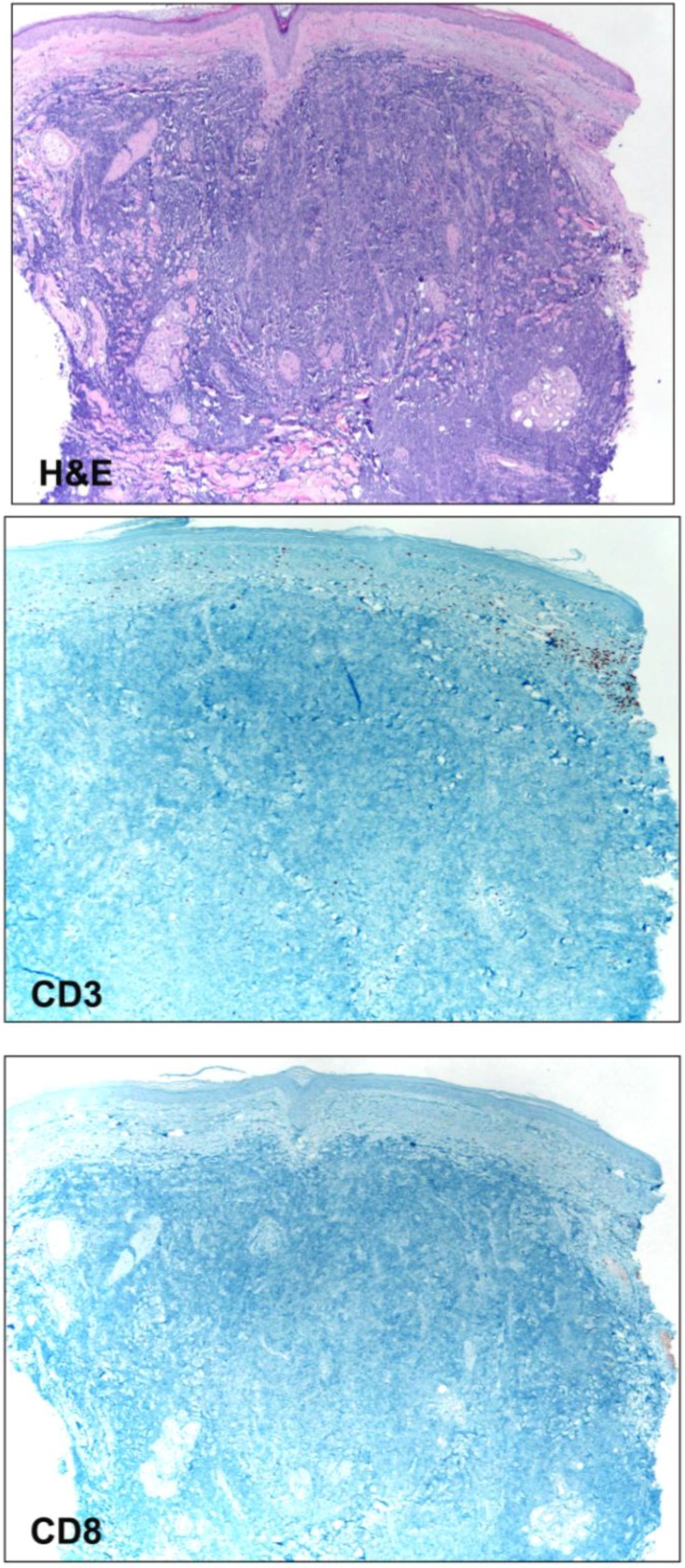
Photomicrographs (4× magnification) of a cutaneous Merkel cell carcinoma displaying sheets of malignant cells (H&E) throughout the dermis. CD3 and CD8 immunostains highlight T-cells within the papillary dermis, but outside of the tumor. No intratumoral lymphocytes are present.

In many solid tumors, including cutaneous melanoma, the presence of tumor infiltrating lymphocytes (TILs) is associated with a better prognosis, supporting a role for the immune response in regulating clinical course [24,25,26]. Several studies have attempted to determine whether specific histologic features of MCC tumors have prognostic significance. Although results have been variable, along with tumor size, the histologic feature found to be most commonly associated with prognosis has been TILs (Table 1). The characterization of TILs has varied but has, for the most part, paralleled the “absent,” “nonbrisk” and “brisk” histologic categories applied in cutaneous melanoma [24]. Immunohistochemical methods identifying specific T cell subsets have also been applied (Figure 1). In studies reported by Andea *et al*. and Llombart *et al*., TILs were associated with improved survivals in univariate but not multivariate analyses [27,28]. Sitho *et al*. reported that a high number of CD3^+^ TILs was associated with better survival in a multivariate analysis [29]. Paulson *et al*. determined that TILs were not an independent prognostic factor when assessed by standard histology, whereas intratumoral, but not peritumoral, CD8^+^ lymphocyte infiltration was, both univariate and multivariate analyses [30]. The observation that intratumoral and not peritumoral lymphocytes are relevant for outcome has also been reported for other cancers, such as ovarian and colon (Figure 2) [31,32]. In contrast, Mott et al. found an association between heavy lymphocytic infiltration of MCC and a poor outcome [33].

**Table 1:** Studies of TILs in MCC.

Study	N	TIL Characterization	Survival	Comment
Mott *et al*. 2004 [33].	25	Minimal/moderate = 17 (68%)	“Heavy” infiltration was associated with poor prognosis in multivariate analysis.	Depth of invasion was associated with poor prognosis in multivariate analysis.
		Heavy = 8 (32%)		
Llombart *et al*. 2005 [28].	20	Absent = 8 (40%)	“Absent” was an independent prognostic factor of DFI in multivariate analysis.	Tumor size>30 mm, stage II, and the presence of >50% of Ki67+ tumor cells were found to be prognostic indicators of disease free interval in univariate analysis.
		Discontinuous = 12 (60%)		
		Continuous = 0		
Andea *et al*. 2008 [27].	156	Absent = 81 (53%)	“Present nondense” plus “Dense” was associated with longer survival in univariate analysis.	Nodular growth pattern, low tumor depth, and absence of lymphovascular invasion were associated with longer survival on multivariate analysis.
		Present nondense = 55 (36%)		
		Dense = 17 (11%)		
Paulson *et al*. 2011 [30].	130	Not identified = 44 (34%)	TILs were associates with better prognosis on univariate but not multivariate analyses.	Intratumoral CD8+ was independently associated with improved survival in multivariate analysis.
		Non-brisk/Brisk = 86 (66%)		
		CD8+ infiltrates (IHC) scored on a 0 to 5 scale		
Sihto *et al*. 2012 [29].	116	Numbers of intratumoral CD3^+^, CD8^+^, CD16^+^, FoxP3^+^, and CD68^+^ cells (IHC) per 1 high power field	High CD3^+^, CD8^+^, or FoxP3^+^ cells, and high CD8^+^/CD4^+^ or FoxP3^+^/CD4^+^ ratios, were significantly associated with favorable overall survival.	Although the numbers of T cells are generally higher in MCPyV-positive than in MCPyV-negative MCC, high intratumoral T-cell counts are also associated with favorable survival in MCPyV-negative MCC.

The relationship of TILs in MCC to MCPyV infection is not well established. Sihto *et al*. found that high numbers of several types of TILs, including T cells (CD3^+^), cytotoxic T cells (CD8^+^), helper T cells (CD4^+^), regulatory T (Treg) cells (FoxP3^+^), and natural killer (NK) cells (small CD16^+^ cells) were linked with presence of MCPyV DNA. There was, however, no evidence that TILs were restricted to the subset of patients whose tumor harbored MCPyV DNA. Patients whose tumor contained a high number of tumor-infiltrating T cells had favorable survival, even when the tumor did not harbor MCPyV DNA, and both high intratumoral T-cell count and presence of MCPyV DNA were independent prognostic factors in a multivariate analysis. These results suggested that MCPyV infection enhances TIL infiltration, but other factors also maintain the host antitumor response. Furthermore, they suggested that a favorable prognosis of MCCs with TILs is not explained by the presence of MCPyV alone [29].

That a high tumor FoxP3^+^ Treg cell count was associated with favorable outcome in the studies of Sitho *et al*. is of interest, since Treg cells are generally implicated in the suppression and not the promotion of antitumor immunity [29]. In fact, patient survival has been shown to correlate negatively with tumor FoxP3^+^ Treg cell infiltration in small-cell lung cancer [34]. Studies in other types of human cancer have been mixed, however, linking high FoxP3^+^ cell counts with either favorable or unfavorable survival, and a recent meta-analysis found no association between tumor Treg cell infiltration and survival [35]. Tumor infiltrating macrophages, which have been associated with the suppression of antitumor immunity and a poor outcome in several cancers, including cutaneous melanoma [36], have also been identified in MCC tumors (Figure 1). Like TILs, the infiltration of macrophages into MCC tumors has been associated with MCPyV; CD68^+^ and CD163^+^ tumor infiltrating macrophages, however, were not significantly associated with survival [29]. Myeloid-derived suppressor cells, a heterogeneous collection of immature myeloid cells with immune suppressive function, have been implicated in promoting the progression of several cancers [37]. Whether these cells are operational in MCC has not been reported.

### 3.2. Tumor Immune Transcripts

Although limited by the heterogeneity of the tissues examined, several groups have applied molecular profiling techniques to examine intratumoral immune responses [38]. Paulson *et al*. examined gene expression profiles in MCC tumors and found those of patients considered to have a favorable prognosis, namely an absence of nodal disease at presentation and an absence of progression at 24 months, were enriched for immune response genes, especially those expressed by CD8^+^ lymphocytes [30]. Prominently represented were genes encoding components of cytotoxic granules (granzymes A, B, H, and K), chemokines (CCL19), lymphocyte activation genes (SLAMF1 and NKG2D), and α chain of the CD8 receptor (CD8a). No association was found between these and tumor MCPyV status. Intratumoral cytokine transcripts were also reported by Arany *et al.* [39]. There was a lack of cytokine transcripts, such as *IL2* and *IFNG*, typical for a specific, T cell-mediated immune response. However, several cytokine transcripts were produced, such as *IL12*, that are required for the activation of this response. Epithelial genes associated with proliferation, such as c-*myc*, cdc 2 kinase, *E2F*, proliferating cell nuclear antigen, *TP53*, *RB*, CK5, and CK10, were also highly expressed.

### 3.3. Spontaneous Regression

Although the mechanism for this phenomenon is uncertain, that the immune response influences progression of MCC is also supported by observations of spontaneous regression. It is difficult to determine the incidence of spontaneous regression of any cancer, primarily because treatment is usually initiated soon after diagnosis. Based on reviewing published reports, which are limited by reporting biases, the estimated rate of regression in MCC is 1.4%, which is very low [40]. Approximately 10% of patients diagnosed with MCC, primarily elderly men, will present with an unknown primary [41,42]. There are data that patients with an occult primary tumor may have better disease-free and overall survivals compared to those without an occult primary tumor, suggesting that resolution of the primary tumor may be an immune-mediated phenomenon that persists when the tumor arises elsewhere [43,44]. Spontaneous regression of an established MCC has only rarely been reported, less than 20 cases, about half of which were metastatic lesions [45]. It appears to be more common in women, in nodal site, when the primary site is the head or neck, and after biopsy or fine needle aspiration. The “abscopal” regression of untreated lesions in a patient undergoing radiation, also considered to be an immune mediated phenomenon, has also been reported [46]. Of note, spontaneous regression can be sustained.

Studies of primary and metastatic MCC that were observed to regress have demonstrated infiltration by immune cells, including CD4^+^, CD8^+^, and CD3^+^ T cells and macrophages [47,48,49]. An examination of tumors that subsequently partially or completely regressed after biopsy found a significant increase in T cells compared to tumors that did not regress [50]. Given that intratumoral infiltration of CD8^+^ T cells was associated with a favorable prognosis, whether diagnostic biopsy induces intratumoral CD8^+^ T cells in MCC was examined; it did not [51]. Lymph nodes draining areas of spontaneous cutaneous regressions have also been examined. They have been enlarged with extensive fibrosis and accumulation chronic inflammatory cells including many T cells and macrophage, suggesting a previous immune-mediated response there, perhaps to metastatic foci [45,48,52,53]. Spontaneous regression has been observed in other virus-associated tumors [54,55,56]. That an immune response to the MCPyV may be operational in the regression of MCC has been speculated but not established [57].

### 3.4. Paraneoplastic Autoimmune Syndromes

Several cancers have been associated with paraneoplastic autoimmune syndromes caused by activation of an immune response specific for self antigens expressed on tumor cells. These too support the immunogenicity of tumors; and although their impact is not established, there is evidence that the presence of an autoimmune paraneoplastic syndrome can confer a survival advantage in small-cell lung cancer [58]. As with other cancers of neuroendocrine derivation, paraneoplastic neurologic disorders have been observed in MCC. Antibodies to cerebellar nerve fibers have been reported in a patient with paraneoplastic cerebellar ataxia associated with MCC [59]. Anti-Hu antibodies have been reported in a MCC patient with painless proximal muscle weakness [60] and in a patient with progressive sensorimotor neuropathy, autonomic neuropathy with gastroparesis, and encephalopathy with cognitive decline [61]. Novel antibodies reactive to filamentous structures in brain and cerebellum were found in a patient with MCC and paraneoplastic brainstem encephalitis [62]. Lambert-Eaton myasthenic syndrome has also been associated with MCC [63]. How the presence of a paraneoplastic syndrome affects the clinical course of MCC course is not known.

### 3.5. Reduction of Immune Suppression

The evidence that the reduction or withdrawal of immune suppression plays a role in the treatment of MCC is, for the most part, anecdotal but further support a role for immune surveillance in association with this rare malignancy. There are case reports of MCC regression in response to withdrawal of immune suppressive therapeutics such as azathioprine and cyclosporine [64,65]. Nonetheless, there is a general consensus that reduction or withdrawal of immune suppression in high-risk transplant-associated skin cancers warrant consideration, particularly in situations where the allograft is not lifesaving (e.g., renal). Several studies have shown a lower incidence of malignancies, including solid tumors of all types, when inhibitors mammalian target of rapamycin, which have been shown to have anti-viral properties, are used alone or in combination with standard agents [66]. How specific immune suppressive drugs influence MCC development is not known.

## 4. MCPyV Immune Response

### 4.1. Humoral

Circulating antibodies against MCPyV viral capsid proteins are present in most patients with MCC and in approximately 50% of children under 15 and up to 80% of healthy people over 50 years of age in the general population. Although the prevalence of antibodies to viral capsid proteins in the general population is high, all studies have found that titers of IgG antibodies to MCPyV vial capsid proteins are higher in patients with MCC [12,67,68,69,70,71]. As noted, MCC tumor cells do not express viral capsid proteins, suggesting that the higher titers are not attributable to increased viral capsid antigen production by tumor cells. A positive correlation between antibody titers and viral load has been observed for other viral infections, including other polyomavirus infections [72,73,74,75,76]. MCPyV DNA levels in cutaneous swabs from MCC patients are significantly higher than controls, and a positive correlation between MCPyV capsid antibody titers and MCPyV DNA levels in skin biopsies has been observed [8,77,78]. Thus, high antibody titers in MCC patients may be the result of poorly controlled MCPyV infection and high viral loads, perhaps promoted by an immune deficiency or an aberrant immune response to viral infection.

Compared to antibodies to viral capsid proteins, antibodies to MCPyV T-antigens are more specifically associated with MCC. T-antigens are not present in viral particles. They are only expressed after viral entry into host cell nuclei, and likely only elicit an antibody response when released by dying infected cells [79]. In approximately 40% of patients with MCC, serum IgG antibodies that recognize a portion of T-antigen shared between LT-Ag and ST-Ag can be identified. These antibodies are rarely detected in the general population (<1%). Importantly, antibody titers correlate strongly with the presence of MCPyV DNA and the expression of T-antigens in MCC tumors. Moreover, titers decrease after successful treatment, and a rising titer in a previously treated patient has been shown to herald disease progression [71]. Whether following T-antigen titers may be used clinically to determine whether treatment is successful or to detect a recurrence requires further study.

Whether humoral responses modulate clinical course is not known. Although antibodies recognizing MCPyV capsid and T-antigens are associated with MCC, they obviously do not protect against disease development. Higher anti-MCPyV capsid antibody titers were associated with a better disease-free survival in one study [80]. Whether this indicates the influence of an antitumor immune response remains unclear. It may reflect MCPyV burden. As noted, patients with MCPyV-negative MCC tumors tend to do less well, perhaps on the basis of altered cellular intrinsic growth mechanisms. The limited serologic data from patients with MCPyV-negative MCC tumors suggest, however, that the majority of these patients have been exposed to MCPyV [70], and in many patients antibody titers can be very high, similar to patients with MCPyV-positive MCC [67]. This suggests the possibility that selection of less immunogenic, more aggressive, MCPyV-negative MCC tumor subclones has occurred in these patients. Antibodies against autologous tumor cell proteins in patients with small-cell lung cancer are associated with improved survival [81]. That antibodies against autologous tumor cell proteins are present in patients with MCC has not been reported.

### 4.2. Cellular

Although humoral response can contribute antitumor activity, the generation of cellular immunity is considered central to tumor elimination. T-cell responses have been described to occur against other polyomaviruses and to other cancer-associated viruses, such as human papilloma (HPV) and hepatitis C viruses, albeit infrequently [82,83,84,85]. That anti-MCPyV antibodies are IgG implies the presence of MCPyV-specific CD4^+^ T-cell help. Compared to MCPyV-seronegative, the CD4^+^ T cells from seropositive volunteers have been shown to respond with significantly higher proliferation and interferon-(IFN-) γ, IL-10, and IL-13 production when stimulated with MCPyV capsid protein [86]. Iyer *et al.* reported the presence of MCPyV-reactive T cells in MCC patients [87]. They isolated virus-reactive CD8^+^ or CD4^+^ T cells from MCPyV-positive but not from virus-negative MCC tumors. MCPyV-specific T-cell responses were also detected in the blood of MCC patients (14 of 27) and control subjects (5 of 13). These T cells recognized a broad range of peptides derived from the capsid and T-antigen and mediated MHC class I (HLA-A*2402) restricted lysis of cells expressing the LT-Ag. Whether cellular responses modulate clinical course of MCC is not known. Virus-specific CD8^+^ T cells were markedly enriched among TILs as compared with blood in one patient, as determined by a HLA-LT-Ag tetramer, implying intact T-cell trafficking into the tumor. Although tetramer-positive CD8^+^ T cells were detected in the blood, these cells failed to produce IFN-γ when challenged *ex vivo* with LT-Ag peptide, suggesting non-responsiveness. The findings to date, however, do suggest that MCC tumors often develop despite the presence of T cells specific for MCPyV T-antigens. Little is known about the role of the NK cells in the immune defense against MCPyV, but NK cells and also γδ T cells have a protective role against polyomavirus-induced tumors in some mouse models [88].

## 5. Immunotherapy

### 5.1. Cytokines

Several cytokines have been effectively applied in cancer therapy. Because of their immunomodulatory, antiviral, and antiproliferative effects, interferons have been used to treat many malignancies. IFN-α is used in the adjuvant setting to treat patients with resected, high-risk cutaneous melanoma. It is also used to treat hepatitis B and C. The antiviral activity of IFN-α and IFN-β, both type I IFNs, has been demonstrated against polyomaviruses [89,90]. Type I IFNs have also been shown to inhibit MCC cell lines *in vitro*; type II IFN-γ is far less effective [91,92]. The inhibitory effect of type I IFNs on MCPyV-positive MCC cell lines *in vitro* was associated with a reduced expression of the LT-Ag as well as an increased expression of promyelocytic leukemia protein, which is known to interfere with the function of the LT-Ag. In addition, the intra-lesional application of IFN-α resulted in a regression of MCPyV-positive but not negative MCCs *in vivo* in a mouse xenograft model [92].

The systemic administration of type I IFNs has been reported to have antitumor activity in patients with MCC. There are case reports of tumor regression with IFN-alfa-2a at 3 million International Units (MIU) subcutaneously three times a week [93] and with IFN-β subcutaneously at 3 MIU per day [94]. Two patients treated in another study with recombinant IFN-alfa-2a at a daily dose of 6 MIU intramuscularly for 8 weeks, and 3 times weekly thereafter did not respond [95]. Two patients treated with recombinant IFN-alfa-2b at 3 MIU daily in another study also did not respond [96]. Whether dosing of the IFNs was optimal in the patients—3 MIU is considered to be a low dose—that did not respond was not clear, particularly in light of the biological heterogeneity of MCC outlined above. IFN has also been injected intra-lesionally in a patient [97]. How the presence of MCPyV influences responses is not known.

There are case reports of the potential antitumor activity of other immune regulatory cytokines in MCC. Two patients were treated with intra-lesional injection of natural human tumor necrosis factor (TNF) [98]. Complete tumor responses were achieved in both without causing ulceration or scarring. Three patients with MCC responded to treatment with hyperthermic isolated limb perfusion with TNF-α, IFN-γ, and melphalan [99]. Sustained regression of metastatic MCC with treatment of HIV infection in patient treated with subcutaneous interleukin-(IL-)2 in conjunction with antiretrovirals (stavudine, lamivudine, and ritonavir) [100]. Patients with local and distant MCC will be enrolled in a Phase II clinical trial designed to assess the clinical and biologic effects of increased local expression of IL-12 protein in the MCC tumor microenvironment following treatment with OMS ElectroImmunotherapy [101].

### 5.2. Adoptive Cellular

One method of dealing with immune cell deficiencies in patients with cancer is to infuse immune cells generated *ex vivo*. A variety of immune effector cells have been generated for adoptive cellular therapy and tested in clinical trials. A recent case report has described the use autologous polyclonal antigen-specific CD8^+^ T cells targeting the HLA A*2402-restricted epitope of the MCPyV T-Ag oncoprotein in a patient with MCC. *Ex-vivo* cultures were supplemented with IL-21, which promotes cytolytic T lymphocyte survival. To induce tumor MHC class I up-regulation, IFN-β was injected intra-lesionally in one of three lesions prior to the first infusion of polyclonal MCPyV-specific T cells. Four weeks later, a single dose of radiation to detectable disease was followed by a second T-cell infusion. Both infusions were followed by low-dose IL-2 administered systemically. Treatment was well tolerated. Four weeks after the first infusion, lesions were noted to regress, and eight weeks after the second infusion, all lesions had regressed by an average of 62% with complete disappearance of the metastatic lesion injected with IFN-β. Infused T cells were observed to persist for at least 6 weeks and to maintain function, as assessed by secretion of IFN-γ, TNF-α and IL-2 in response to T antigen. The contribution of the cells infused to the antitumor effects is difficult to determine, as IFN-β, IL-2, and radiation were also administered. Nonetheless, the results do support further study, and extension of this approach to other patients with MCC using a broader array of MHC-restricted MCPyV epitopes is planned [102].

### 5.3. Topical

Topical immune modulators have been applied to treat a variety of skin cancers. Contact sensitizers such as dinitrochlorbenzol form stable conjugates with proteins in the epidermis and elicit cytokine production by skin cells. Sensitization has been shown to occur within the regional lymph nodes, where specific T cells come into contact with the bound dinitrochlorbenzol. A patient with multiple local and regional MCC metastases was reported to respond with complete regression to four weekly treatments of topically applied dinitrochlorbenzol [103]. A biopsy of a regressing lesion showed few remaining cytokeratin-20-positive tumor cell complexes in the mid to lower dermis surrounded by a dense infiltration consisting of T cells and macrophages. Imiquimod is a synthetic molecule that activates Toll-like receptors 7 and 8, the natural ligand of which is pathogen-derived, single-stranded RNA. Both innate and adaptive immune systems are activated by application of imiquimod. A case report suggests the possible effective use of imiquimod, combined with radiation therapy, for MCC [104,105].

### 5.4. Vaccines

Therapeutic vaccines have been an attractive approach to many refractory malignancies. Preclinical studies have supported their use in MCC. A DNA vaccine encoding the truncated LT-Ag was able to produce antitumor effects against LT-Ag-expressing tumor cells in a mouse model. Efficacy was found to be predominantly a result of CD4^+^ T cells, although NK cells and CD8^+^ T cells did contribute to the therapeutic effects [106]. Linking a damage-associated molecular protein, calreticulin, to the LT-Ag in a DNA vaccine construct enhanced the generation of therapeutic CD8^+^ T cells [107]. Merkel cells and the majority of MCC express Ep-CAM, gangliosides, and MUC 1, which represent tumor-associated antigens currently being targeted via vaccine production and/or monoclonal antibody treatment strategies in other cancers [108,109,110,111,112]. Whether these will translate into effective treatment strategies for MCC remains to be determined.

## 6. Conclusions and Future Directions

Despite an increase in clinical and experimental data, how the immune response influences MCC development and progression remains poorly understood. It is highly likely that the immune system plays a central role in preventing and controlling MCC, as patients who are immunosuppressed for various reasons have an increased risk. There is evidence that high intratumoral T-cell counts and immune transcripts are associated with favorable survival. Spontaneous regressions also implicate immune effector mechanisms in MCC control. Immunogenicity is also supported by observation of autoimmune paraneoplastic syndromes. Case reports suggest that immune modulation, including reduction of immune suppression, can result in MCC regression.

The relationships between MCPyV infection, the immune response, and MCC outcome also remain poorly understood. Circulating serum antibodies against MCPyV T antigen and capsid proteins are present in most individuals. MCPyV-reactive T-cells can be detected in the blood of both MCC patients and control subjects. These observations suggest that MCC often develops despite the presence of a humoral and cellular immune response against MCPyV. In the future, MCPyV-targeted immunotherapies may be developed. A better understanding on how MCPyV evades the immune response will be necessary, however, to develop effective treatments. Recent studies have focused on the production of microRNA by MCPyV that can mediate immune invasion [113]. Methods are being developed to modulate microRNA clinically [114]. It is not clear that a preventive vaccine targeting MCPyV will be clinically useful, as are preventive vaccines targeting the HPV and hepatitis oncoviruses. The link of MCC to MCPyV will need to be more convincingly established, as will the prevalence of morbidity from associated cancer and/or potentially other diseases.

Not all MCC tumors have been associated with MCPyV. Although the numbers of intratumoral T cells are generally higher in MCPyV-positive than in MCPyV-negative MCC, high intratumoral T-cell counts are also associated with favorable survival in MCPyV-negative MCC, suggesting that responses to non-MCPyV, tumor-associated antigens are operational. The identity of these antigens, however, is not currently known. Further characterization of tumor and TILs could lead to identification of these antigens and novel MCC targeted immunotherapies.

Improved understanding of the mechanisms by which T-cell activation is regulated has led to the identification of “checkpoint” molecules that inhibit T-cell reactivity. Ipilimumab, an antibody that blocks cytotoxic T-lymphocyte antigen-4 (CTLA-4), an inhibitory molecule present on activated T cells, is used to treat patients with metastatic melanoma [115]. In a phase II trial in patients with small-cell lung cancer, there was evidence that ipilimumab improved progression-free survival [116]. Programmed death (PD)-1 is another immune inhibitory molecule, and initial clinical testing of a blocking antibody to the PD-1 receptor and to the PD-1 ligand has shown encouraging results [117,118]. Immune stimulatory molecules, such as 41-BB, CD40, and OX-40, are also targets, and early-phase clinical trials of activating antibodies to these have shown that they can mediate tumor regression [119]. The clinical activity of checkpoint modifiers in MCC has not, to date, been reported. Clinical trials are likely to be conducted, but more data on what checkpoints are operational in MCC are needed.

The observations supporting a role for host immunity in the incidence and course of MCC suggest that immune modulating therapies could be highly effective in treating MCC. To date, however, there are only a few reports on the activity of immunotherapies in MCC. MCC is often approached therapeutically with approaches developed for the far more common skin cancer, melanoma, and for the far more common neuroendocrine carcinoma, small-cell lung. Given the emerging role of MCPyV in MCC, these extrapolations are tenuous. The incidence of MCC has been increasing [1]. The stage is now set for more specific approaches.
